# Reduced concentrations of limestone and monocalcium phosphate in diets without or with microbial phytase did not influence gastric pH, fecal score, or growth performance, but reduced bone ash and serum albumin in weanling pigs

**DOI:** 10.1093/tas/txab115

**Published:** 2021-07-05

**Authors:** L Vanessa Lagos, Su A Lee, Mike R Bedford, Hans H Stein

**Affiliations:** 1 Division of Nutritional Sciences, University of Illinois, Urbana, IL, USA; 2 Department of Animal Sciences, University of Illinois, Urbana, IL, USA; 3 AB Vista, Marlborough, UK

**Keywords:** bone ash, dietary Ca and P, fecal score, gastric pH, pigs, phytase

## Abstract

An experiment was conducted to test the hypothesis that reducing limestone and monocalcium phosphate in diets for weanling pigs by lowering the concentration of Ca and P or by including microbial phytase in the diet will reduce stomach pH and fecal score and will improve growth performance of pigs. A total of 160 weanling pigs (5.75 ± 1.04 kg) were allotted to four corn-soybean meal-based diets in a completely randomized design with five pigs per pen. Diets for phase 1 (d 1 to 15) were formulated using a 2 × 2 factorial design with 2 concentrations of Ca and P (adequate or deficient levels of total Ca and digestible P) and 2 inclusion levels of phytase (0 or 2,000 units/kg feed). Phytase was assumed to release 0.16% total Ca and 0.11% digestible P. Common diets were fed in phases 2 (d 16 to 21) and 3 (d 22 to 35). Fecal scores were recorded in phase 1 and on d 15, gastric pH was measured and a blood sample and the right femur were collected from one pig per pen. Growth performance data were recorded within each phase. Results indicated that in phase 1, at deficient dietary Ca and P, pigs fed the diet with phytase had greater (*P* < 0.05) average daily gain (ADG) and gain to feed (G:F) compared with pigs fed the diet without phytase, but in diets with adequate levels of Ca and P, no effect of phytase inclusion was observed (interaction, *P* < 0.05). Without phytase, pigs fed the diet with deficient Ca and P had reduced (*P* < 0.05) G:F compared with pigs fed the diet with adequate Ca and P, but if phytase was included, there was no effect of Ca and P on G:F (interaction, *P* < 0.05). For phases 2 and 3, and from d 1 to 35, no differences among dietary treatments were observed for ADG or G:F. Bone ash was greater (*P* < 0.05) in pigs fed diets with adequate Ca and P than in pigs fed diets with deficient Ca and P, but no effect of phytase inclusion was observed on bone ash. Concentrations of Ca and P did not affect stomach pH or fecal score, but pigs fed diets with phytase tended (*P* < 0.10) to have reduced stomach pH and fecal score compared with pigs fed diets without phytase. Pigs fed diets with adequate Ca and P had greater (*P* < 0.05) albumin in serum than pigs fed the Ca- and P-deficient diets. In conclusion, phytase inclusion in phase 1 diets may reduce diarrhea, but lowering Ca and P does not reduce stomach pH or fecal score and decreases bone ash, although growth performance during the entire weanling period is not affected.

## INTRODUCTION

Weaning is a critical period for pigs because they are exposed to a variety of stressors including removal from the sow, transportation, interaction with pigs from different litters, and the transition from sow milk to a less digestible diet (Pluske et al., 1997). The change in diet may result in disrupted intestinal barrier function and often causes diarrhea because young pigs have reduced digestive enzymatic activity compared with older pigs (Hedemann et al., 2006). To mitigate the negative effects of weaning on growth performance of pigs, antibiotic growth promoters (AGP) have been used in nursery diets, but restriction on the use of AGP has increased due to the risk of intestinal microbes acquiring resistance to antibiotics (Casewell et al., 2003). As a consequence, direct-fed microbials, prebiotics, phytogenic feed additives, and acidifiers have been investigated as alternatives to AGP (Liu et al., 2018). Dietary acidifiers are used to create an adequate gastric environment that favors pepsin activity (Liu et al., 2018) because weanling pigs lack the ability to secrete sufficient HCl in the stomach to reach a stable low pH for proper digestion of proteins (Suiryanrayna and Ramana, 2015). However, inclusion of limestone and monocalcium phosphate (MCP) in weaning diets may exacerbate problems caused by limited secretion of HCl because these ingredients have a high buffering capacity at pH 3 (Lawlor et al., 2005). Therefore, reducing limestone and MCP in phase 1 diets may result in decreased stomach pH. Inclusion of microbial phytase in diets increases the digestibility of Ca and P and reduces the necessity for MCP and limestone in diets (Selle et al., 2009), which may further contribute to a reduced pH in the stomach. However, data to demonstrate these hypotheses are limited. Therefore, the objective of this experiment was to test the hypothesis that reducing the amount of limestone and MCP in diets for weanling pigs by lowering the concentrations of dietary Ca and P, and (or) by including microbial phytase in the diet, will reduce stomach pH and fecal score and improve growth performance of pigs.

## MATERIALS AND METHODS

The Institutional Animal Care and Use Committee at the University of Illinois reviewed and approved the protocol for the experiment. Pigs used in the experiment were the offspring of Line 359 boars and Camborough females (Pig Improvement Company, Hendersonville, TN).

### Animals, Housing, and Diets

One hundred and sixty weanling pigs with an initial body weight (BW) of 5.75 ± 1.04 kg were randomly allotted to four diets in a completely randomized design. There were five pigs per pen (three gilts and two castrates) and eight replicate pens per diet. Pens had fully slatted floors, a feeder, and a nipple drinker. Feed and water were available at all times. The Experimental Animal Allotment Program (Kim and Lindemann, 2007) was used to allot pigs to experimental diets.

The experiment was conducted for 5 wk. A 3-phase feeding program was used with d 1 to 15 as phase 1, d 16 to 21 as phase 2, and d 22 to 35 as phase 3. Pigs were fed one of four diets during phase 1, whereas a common diet was fed in phases 2 and 3. Therefore, a total of six diets were formulated (Table 1).

**Table 1.** Composition and analyzed values of experimental diets^1^

Item^2^	Phase 1					
Phytase:	0 FTU		2,000 FTU			
Ca and P levels:	Adequate	Deficient	Adequate	Deficient	Phase 2	Phase 3
Ingredient, %						
Ground corn	42.42	45.91	43.85	46.80	42.01	51.55
Soybean meal, 48% crude protein	22.00	22.00	22.00	22.00	28.00	32.00
Lactose	15.00	15.00	15.00	15.00	15.00	10.00
Soy protein concentrate	8.00	8.00	8.00	8.00	8.00	-
Enzyme-treated soybean meal	2.50	2.50	2.50	2.50	-	-
Spray-dried plasma protein	3.00	3.00	3.00	3.00	-	-
Soybean oil	3.00	1.26	2.29	0.82	3.00	3.00
Ground limestone	1.28	0.72	1.13	0.37	1.19	1.11
Monocalcium phosphate	1.29	0.13	0.69	-	1.27	0.94
l-Lys HCl	0.26	0.25	0.26	0.25	0.31	0.38
dl-Met	0.17	0.16	0.16	0.15	0.17	0.15
l-Thr	0.08	0.07	0.08	0.07	0.10	0.12
Sodium chloride	0.85	0.85	0.85	0.85	0.80	0.60
Vitamin-mineral premix^3^	0.15	0.15	0.15	0.15	0.15	0.15
Phytase concentrate^4^	-	-	0.04	0.04	-	-
Total	100.00	100.00	100.00	100.00	100.00	100.00
Calculated values^5^, %						
Total Ca	0.83	0.42	0.67	0.26	0.80	0.70
Total P	0.66	0.43	0.54	0.40	0.64	0.56
STTD P	0.43	0.22	0.32	0.20	0.40	0.33
Analyzed values						
Gross energy, kcal/kg	3,990	3,947	3,982	3,966	3,973	3,968
Dry matter, %	89.41	88.84	89.46	89.23	88.75	88.62
Ash, %	6.33	4.63	5.80	4.54	5.63	5.27
Crude protein, %	21.50	22.21	21.87	22.58	20.34	18.58
AEE, %	4.81	2.88	4.20	2.61	4.48	4.35
Amino acids, %						
Arg	1.49	1.42	1.50	1.39	1.38	1.30
His	0.59	0.58	0.60	0.58	0.54	0.50
Ile	1.02	0.98	1.03	0.98	0.95	0.89
Leu	1.87	1.83	1.90	1.83	1.72	1.58
Lys	1.60	1.53	1.60	1.63	1.50	1.40
Met	0.51	0.44	0.48	0.42	0.43	0.40
Phe	1.13	1.09	1.15	1.09	1.04	0.97
Thr	1.00	0.94	1.03	0.97	0.95	0.91
Trp	0.29	0.28	0.28	0.28	0.27	0.25
Val	1.18	1.14	1.19	1.13	1.06	0.96
Ca, %	0.91	0.47	0.65	0.22	0.90	0.79
P, %	0.64	0.46	0.56	0.38	0.69	0.61
Phytate^6^, %	0.90	0.93	0.91	0.93	0.95	0.93
Phytate bound-P^7^, %	0.25	0.26	0.26	0.26	0.27	0.26
Non-phytate P^8^, %	0.39	0.20	0.30	0.12	0.42	0.35
Phytase activity, FTU/kg	< 70	< 70	1,400	1,400	< 70	< 70

^1^Phase 1, phase 2, and phase 3 diets were formulated to have the following quantities of net energy (NE; kcal/kg) and amino acids (expressed as standardized ileal digestible; %): NE, 2,518, 2,496, and 2,498; Lys, 1.41, 1.35, and 1.23; Met, 0.46, 0.45, and 0.41; Thr, 0.83, 0.80, and 0.74; Trp, 0.26, 0.24, and 0.22, respectively.

^2^AEE = acid hydrolyzed ether extract; FTU = phytase units per kilogram of feed; STTD = standardize total tract digestible.

^3^The vitamin-micromineral premix provided the following quantities of vitamins and micro minerals per kg of complete diet: vitamin A as retinyl acetate, 11,150 IU; vitamin D_3_ as cholecalciferol, 2,210 IU; vitamin E as dl-alpha tocopheryl acetate, 66 IU; vitamin K as menadione dimethylprimidinol bisulfite, 1.42 mg; thiamin as thiamine mononitrate, 1.10 mg; riboflavin, 6.59 mg; pyridoxine as pyridoxine hydrochloride, 1.00 mg; vitamin B_12_, 0.03 mg; d-pantothenic acid as d-calcium pantothenate, 23.6 mg; niacin, 44.1 mg; folic acid, 1.59 mg; biotin, 0.44 mg; Cu, 20 mg as copper sulfate; Fe, 125 mg as ironsulfate; I, 1.26mg as ethylenediamine dihydriodide; Mn, 60.2 mg as manganous sulfate; Se, 0.30 mg as sodium selenite and selenium yeast; and Zn, 125.1 mg as zinc sulfate.

^4^The phytase concentrate contained 5,000 units of phytase/g (Quantum Blue, AB Vista, Marlborough, UK).

^5^Phytase was assumed to release 0.16% total Ca and 0.11% STTD P.

^6^Phytate values in the diets were calculated from analyzed phytate in the ingredients.

^7^Phytate-bound P was calculated by multiplying the phytate by 0.282 (Tran and Sauvant, 2004).

^8^Non-phytate P was calculated as the difference between total P and phytate-bound P.

The four diets in phase 1 were based on corn and soybean meal and were formulated using a 2 × 2 factorial design with two concentrations of Ca and P (adequate or deficient levels of total Ca and standardized total tract digestible (STTD) P) and two inclusion levels of microbial phytase (0 or 2,000 phytase units per kilogram of feed (FTU)). Diet 1 was formulated based on the NRC (2012) requirement for total Ca (0.83%) and STTD P (0.43%). Diet 2 was formulated with 50% of the NRC (2012) requirement for total Ca (0.42%) and STTD P (0.22%). Diet 3 was similar to diet 1 with the exception that 2,000 FTU of microbial phytase (Quantum Blue; AB Vista Feed Ingredients, Marlborough, UK) were included, and the provisions of total Ca and STTD P were reduced by 0.16% and 0.11%, respectively, to account for the expected release of Ca and P by phytase. Diet 4 was formulated as diet 2, but with 2,000 FTU of phytase, and the provision of total Ca was reduced as explained for diet 3, thus the concentration of total Ca was 0.26%. However, STTD P was only reduced to 0.20%, which corresponds to 72% of the NRC (2012) requirement, because that was the value obtained after removal of all MCP in the diet and accounting for the expected release of P by phytase (Table 2). The four diets were formulated to contain identical quantities of net energy, Na, Cl, K, and vitamin D. Phase 2 and 3 diets were formulated to meet requirements for total Ca (0.80% and 0.70%, respectively) and STTD P (0.40% and 0.33%, respectively; NRC, 2012) and no phytase was used in these diets.

**Table 2.** Analyzed composition of ingredients

Item	Corn	Soybean meal	Soy protein concentrate	Enzyme treated soybean meal	Spray-dried plasma protein	Calcium carbonate	Monocalcium phosphate
Gross energy, kcal /kg	3,843	4,162	4,265	4,468	4,813	-	-
Dry matter, %	86.87	88.00	88.80	92.46	91.21	99.95	95.72
Ash, %	1.21	7.17	6.24	8.22	8.46	90.03	81.25
Crude protein, %	6.47	45.84	61.57	54.92	79.13	-	-
AEE^1^, %	3.64	2.43	1.65	2.53	1.05	-	-
Ca, %	0.01	0.31	0.34	0.30	0.10	39.40	16.89
P, %	0.27	0.68	0.76	0.74	1.18	0.07	21.73
Phytate, %	0.82	1.60	1.92	1.72	-	-	-
Phytate-bound P^2^, %	0.23	0.45	0.54	0.49	-	-	-
Non-phytate P^3^, %	0.04	0.23	0.22	0.25	-	-	-

^1^AEE = acid hydrolyzed ether extract.

^2^Phytate-bound P was calculated by multiplying the phytate by 0.282 (Tran and Sauvant, 2004).

^3^Non-phytate P was calculated as the difference between total P and phytate-bound P.

### Sample Collection and Bone Measurements

Pig weights were recorded at the beginning and at the conclusion of each phase. The amount of feed offered was recorded every day and the amount of feed left in the feeders was recorded at the end of each phase. During the initial 15 d, fecal scores were assessed visually every other day using a score from 1 to 5 (1 = normal feces; 2 = moist feces; 3 = mild diarrhea; 4 = severe diarrhea; and 5 = watery diarrhea). On the last day of phase 1, the gilt in each pen with a BW closest to the average BW of the pen was euthanized via captive bolt stunning and a blood sample was collected in vacutainers that contained spray-coated silica to yield blood serum after centrifugation at 1,500 × *g* at 4 ºC for 15 min. Serum samples were frozen at −20 ºC until used for analysis of blood urea nitrogen (BUN), total protein, and albumin. The abdominal cavity of the euthanized pig was opened and pH of gastric contents was measured twice *in situ* by making a small incision for a pH electrode in the stomach cavity. All stomach content was then collected and mixed and *ex situ* pH was measured twice. The right femur was collected and autoclaved at 125 ºC for 55 min and the muscles attached to the bone were removed. Femurs were broken, bone marrow was removed, and bones were dried overnight at 105 ºC. Femurs were then soaked for 72 h in petroleum ether under a chemical hood to remove residual marrow and fat. Bones were then dried at 135 ºC for 2 h and ashed at 600 ºC for 16 h.

### Sample Analysis

Corn, soybean meal, soy protein concentrate, enzyme treated soybean meal, spray dried plasma protein, calcium carbonate, MCP, and diets were analyzed for dry matter by oven drying at 135 °C for 2 h (Method 930.15; AOAC Int., 2019) and for ash by incineration at 600 °C for 2 h (Method 942.05; AOAC Int., 2019). These samples were also analyzed for Ca and P by inductively coupled plasma-optical emission spectrometry (Method 985.01 A, B, and C; AOAC Int., 2019) after wet ash sample preparation (Method 975.03 B(b); AOAC Int., 2019). Corn, soybean meal, soy protein concentrate, enzyme-treated soybean meal, and diets were analyzed for N (Method 990.03; AOAC Int., 2019) using a LECO FP628 (LECO Corp., Saint Joseph, MI) and crude protein was calculated as N × 6.25. These samples were also analyzed for gross energy using an isoperibol bomb calorimeter (Model 6400, Parr Instruments, Moline, IL) and for acid hydrolyzed ether extract (Method 2003.06; AOAC Int., 2019) using 3 N HCl in an Ankom HCl hydrolyzer followed by petroleum ether in an Ankom XT15 extractor (Ankom Technology, Macedon, NY). Corn and soybean products were also analyzed for phytic acid by analytical biochemistry (Ellis et al., 1977). Diet samples were analyzed for amino acids (Method 982.30 E (a, b, c); AOAC Int., 2019) using a Hitachi Analyzer (Model No. L8800; Hitachi High Technologies America, Inc., Pleasanton, CA), and phytase activity was analyzed by the colorimetric enzymatic method (Method AOAC 2000.12; AOAC Int., 2019). Serum samples were analyzed for BUN, total protein, and albumin using a Beckman Coulter Clinical Chemistry AU analyzer (Beckman Coulter, Inc., Brea, CA). The pH of stomach contents was measured using a Benchtop pH meter (Orion Star A111, Fisher Scientific, Waltham, MA) with a pH electrode for semi-solid samples. The pH meter was calibrated using three buffer solutions (4.01, 7.01, and 10.01 pH; Fisher Scientific, Waltham, MA).

### Calculations and Statistical Analyses

The concentration of phytate-bound P in corn and soybean products was calculated by multiplying the analyzed concentration of phytate by 0.282 (Tran and Sauvant, 2004) and non-phytate P was calculated by subtracting the amount of phytate-bound P from total P. Average daily gain (ADG), average daily feed intake (ADFI), and gain to feed ratio (G:F) were calculated for each phase and for the overall experimental period. Bone ash percentage was calculated by dividing the quantity of bone ash by the weight of the fat-free dried bone and multiplying by 100. Diarrhea frequency was calculated by dividing the number of days with fecal score ≥ 3 by the total number of scoring days and multiplied by 100.

Normality of residuals and homogeneity of variances were tested using the INFLUENCE, GPLOT, and UNIVARIATE procedures of SAS (SAS Inst. Inc., Cary, NC). Data were analyzed using the PROC MIXED procedure of SAS. Pen was the experimental unit for growth performance and fecal evaluation, whereas the sacrificed pig in each pen was the experimental unit for stomach pH, bone ash, and blood metabolites. The fixed effects of the model were concentrations of Ca and P, phytase inclusion, and the interaction between concentrations of Ca and P and phytase inclusion. If the interaction was not significant, only main effects were included in the final model. Outliers were determined by plotting the residuals in a quantile–quantile plot against the normal distribution and identifying values that were beyond ± 2.5 standard deviations. The model also included the random effect of replicate. Treatment means were calculated using the LSMEANS statement and means were separated using the PDIFF option of SAS. Statistical significance and tendency were considered at *P* < 0.05 and 0.05 ≤ *P* < 0.10, respectively.

## RESULTS

Pigs remained healthy during the experiment and consumed their assigned diets without apparent health issues. However, one pig fed the diet formulated to be deficient in Ca and P and with 2,000 units of phytase died at the end of phase 1. Values for ADFI in this pen were adjusted as previously described (Lindemann and Kim, 2007). No other pigs died during the experiment. For ADG and G:F in phase 1, an interaction (*P* < 0.05) between concentration of Ca and P and phytase inclusion was observed (Table 3). At adequate levels of Ca and P, no difference was observed for ADG or G:F of pigs fed diets without or with 2,000 FTU of microbial phytase. However, for pigs fed diets with deficient Ca and P, ADG and G:F were greater (*P* < 0.05) if the diet containing phytase was fed rather than the diet without phytase. No difference between concentrations of Ca and P was observed for G:F of pigs fed diets with 2,000 FTU of phytase, but pigs fed non-phytase diets had reduced (*P* < 0.05) G:F if the diet was formulated with deficient concentrations of Ca and P compared with the diet with adequate Ca and P. There was a tendency (*P* < 0.10) for an interaction between dietary Ca and P and phytase inclusion for ADFI with ADFI tending to be reduced for pigs fed the Ca- and P-deficient diet compared with pigs fed the diet with adequate Ca and P if no phytase was used, but the opposite trend was observed if phytase was included in the diet. The BW of pigs at the end of phase 1 was not influenced by dietary treatments. For phases 2 and 3 and for the overall experimental period, no effect of dietary Ca and P concentrations or phytase inclusion in phase 1 diets were observed for growth performance parameters.

**Table 3.** Growth performance of pigs fed diets formulated with adequate or deficient levels of Ca and P (Ca-P) without microbial phytase or with 2,000 phytase units/kg of feed (FTU)^1^

	0 FTU		2,000 FTU			*P*-value		
Item, kg	Adequate	Deficient	Adequate	Deficient	SEM	Ca-P	Phytase	Ca-P × phytase
Phase 1, d 1 to 15								
Initial BW	5.73	5.77	5.75	5.75	0.379	0.960	0.999	0.947
ADG	0.150^ab^	0.112^b^	0.139^ab^	0.176^a^	0.014	0.969	0.077	0.014
ADFI	0.218	0.190	0.196	0.234	0.016	0.758	0.516	0.053
G:F	0.684^a^	0.565^b^	0.705^a^	0.752^a^	0.031	0.252	0.002	0.013
Final BW	7.98	7.45	7.83	8.38	0.556	0.982	0.485	0.343
Phase 2, d 16 to 21								
ADG	0.305	0.294	0.296	0.256	0.032	0.439	0.462	0.655
ADFI	0.421	0.428	0.424	0.430	0.030	0.840	0.933	0.985
G:F	0.733	0.698	0.686	0.589	0.058	0.264	0.190	0.597
Final BW	9.82	9.24	9.53	9.94	0.678	0.901	0.764	0.473
Phase 3, d 22 to 35								
ADG	0.513	0.507	0.488	0.542	0.029	0.420	0.868	0.323
ADFI	0.771	0.746	0.753	0.820	0.044	0.641	0.527	0.300
G:F	0.667	0.682	0.649	0.660	0.014	0.343	0.156	0.896
Final BW	17.00	16.34	16.37	17.53	1.050	0.812	0.795	0.395
Overall, d 1 to 35								
ADG	0.323	0.305	0.308	0.325	0.022	0.984	0.908	0.429
ADFI	0.470	0.455	0.458	0.495	0.028	0.706	0.623	0.354
G:F	0.690	0.669	0.668	0.653	0.019	0.356	0.321	0.896

^a-b^Means within a row lacking a common superscript letter are different (*P* < 0.05).

^1^Data are least squares means of eight observations.

There was no interaction between dietary Ca and P and phytase inclusion for bone ash, stomach pH, fecal scores, or blood metabolites (Table 4). The concentration and percentage of bone ash was greater (*P* < 0.05) in pigs fed diets formulated to be adequate in Ca and P than in pigs fed diets that were deficient in Ca and P. However, no effect of phytase on the concentration or percentage of bone ash was observed. There was no effect of dietary concentration of Ca and P on stomach pH (*in situ*, *ex situ*, or the average). Likewise, there was no effect of phytase on *in situ* stomach pH, but pigs fed diets with 2,000 FTU tended (*P* < 0.10) to have reduced *ex situ* stomach pH and average stomach pH compared with pigs fed diets without phytase. Pigs fed diets with phytase also tended (*P* < 0.10) to have reduced fecal score and diarrhea frequency compared with pigs fed diets without phytase, but no effect of dietary Ca and P on fecal score or diarrhea frequency was observed. There was no effect of dietary phytase on serum concentration of BUN, total protein, or albumin, and the concentration of BUN in serum of pigs fed diets with deficient or adequate Ca and P was not different. However, pigs fed diets with adequate Ca and P tended (*P* < 0.10) to have greater total protein and had greater (*P* < 0.05) albumin concentration in serum than pigs fed diets that were deficient in Ca and P.

**Table 4.** Bone mineralization, stomach pH, and serum metabolites at d 15 and feces evaluation from phase 1 of pigs fed diets formulated with adequate or deficient levels of Ca and P (Ca-P) without microbial phytase or with 2,000 phytase units/kg of feed (FTU)^1^

	Ca and P levels		Phytase, FTU			*P*-value	
Item	Adequate	Deficient	0	2,000	SEM	Ca-P	Phytase
Bone mineralization^2^							
Ash, g	4.92	3.92	4.36	4.48	0.92	0.017	0.771
Ash, %	53.0	49.9	51.9	51.0	0.43	< 0.001	0.147
Stomach pH^3^							
*In situ*	2.73	2.67	2.86	2.53	0.26	0.868	0.377
*Ex situ*	2.90	2.95	3.21	2.64	0.20	0.864	0.058
Average	2.88	2.81	3.10	2.59	0.18	0.788	0.053
Fecal evaluation^2^							
Fecal score	1.88	1.82	1.99	1.71	0.10	0.667	0.060
Diarrhea frequency,^4^ %	25.8	22.7	30.5	18.0	5.04	0.664	0.090
Serum metabolites							
BUN,^5^^,^^6^ mg/dL	14.0	13.8	13.9	13.8	0.92	0.850	0.923
Total protein^5^, g/dL	4.31	4.06	4.18	4.18	0.09	0.052	0.997
Albumin^2^, g/dL	2.51	2.29	2.43	2.38	0.06	0.012	0.533

^1^Data are shown as main effects because the interaction between concentrations of Ca and P and phytase inclusion was not significant (*P* > 0.05).

^2^Data are least squares means of 16 observations.

^3^Data are least squares means of 13 to 16 observations.

^4^Diarrhea frequency = number of days with fecal score ≥ 3 ÷ total number of days × 100.

^5^Data are least squares means of 15 or 16 observations.

^6^BUN = blood urea nitrogen.

## DISCUSSION

The interest in non-antibiotic feed additives for weaning diets has increased in the last two decades due to the risk of continued use of antibiotic growth promoters resulting in development of resistance to antibiotics used for animal and human disease treatment (Wegener, 2003). Among the numerous alternatives to AGP, acidifiers have been widely used in weaning diets because young pigs are believed to have a low ability to produce gastric acid (Kil et al., 2011). Acidifiers have the potential to provide an adequate environment in the stomach through pH reduction for proper nutrient digestion (Kil et al., 2011). However, diet composition also plays an important role because some feed ingredients have a high capacity to bind acid in the stomach of pigs, and inclusion of these ingredients in weaning diets may result in increased gastric pH (Lawlor et al., 2005). The buffering capacity for an aqueous solution is defined as the amount of acid required to influence a pH unit (Urbansky and Schock, 2000). Thus, commonly used sources of Ca and P such as limestone and MCP have a greater buffering capacity at pH 3 and 4 than ingredients originating from animals or plants including milk products, cereal grains, and plant proteins (Jasaitis et al., 1987; Lawlor et al., 2005). Indeed, it was hypothesized that a reduced concentration of ash in diets for weanling pigs can reduce diarrhea occurrence, but this may also affect growth performance and bone development as a result of mineral deficiencies (Bolduan et al., 1988).

Diets used in this experiment were formulated using NRC (2012) values for Ca and P in corn, soybean products, spray-dried plasma protein, calcium carbonate, and MCP, and the analyzed values for these ingredients were close to those used in diet formulation. Therefore, the small differences observed between calculated and analyzed values in diets for Ca are likely a consequence of feed particle segregation within diets that results in wider analytical ranges for Ca than for P (Jones et al., 2018). Ingredient and diet samples were analyzed for Ca and P in triplicate. The reason phytase inclusion was greater than the standard level of 500 FTU, which is often used in the industry, was that previous data indicate beneficial effects on growth performance of newly weaned pigs of including phytase above 1,000 FTU in weanling diets (Moran et al., 2017, 2019).

The observation that regardless of inclusion of microbial phytase, final BW, ADG, or ADFI of pigs were not influenced by the concentration of Ca and P in diets during phase 1 concurs with data from Létourneau-Montminy et al. (2010) indicating that the level of Ca and P does not affect growth performance of pigs during the phase 1 period. Schlegel and Gutzwiller (2017) also reported that growth performance of weanling pigs was not affected by the concentration of Ca when three different levels of Ca were used in diets with similar concentration of P and fed for 2 wk. The lack of differences in growth performance parameters in phases 2 and 3 indicates that pigs are able to recover from Ca- and P-deficient diets, which concurs with data from weanling pigs evaluated for 25 d after a 10-d period with a low Ca and P diet (Létourneau-Montminy et al., 2010).

Although inclusion of 2,000 FTU of phytase did not influence growth performance of pigs fed diets formulated to be adequate in Ca and P, the observation that pigs fed diets with deficient levels of Ca and P had improved ADG and G:F if phytase was used indicates that phytase ameliorates the negative effects of low Ca and P in diets. However, in the diet with phytase and deficient concentrations of Ca and P, P was included at 72% of the NRC (2012) requirement, and the concentrations of limestone and MCP were the lowest among treatments. Under commercial conditions, inclusion of phytase above 1,000 FTU in diets with no reduction of Ca and P resulted in improved growth performance of pigs compared with diets without phytase when fed for 10 d after weaning (Moran et al., 2017, 2019).

The observation that reducing concentrations of Ca and P in phase 1 diets results in decreased concentration and percentage of bone ash concurs with published data from weanling pigs (Létourneau-Montminy et al., 2010; Schlegel and Gutzwiller, 2017) and broiler chickens (Walk et al., 2012). The requirement for Ca and P to maximize bone ash of pigs is greater than the requirement needed to maximize growth performance (Lagos et al., 2019), which explains the response in bone ash even though little effect of reduced dietary Ca and P during the first 2 wk post-weaning was observed for growth performance parameters. Bone ash was not measured after an adequate diet was fed in phases 2 and 3 and it is, therefore, not known if pigs are able to recover bone ash after the depletion in phase 1. However, Létourneau-Montminy et al. (2010) reported that after a 25-d repletion period, pigs fed Ca- and P-deficient diets for 10 d post-weaning tended to have lower bone ash than pigs fed diets with adequate levels of Ca and P.

Results from this experiment reject the hypothesis that lowering the concentration of Ca and P in phase 1 diets reduces stomach pH of pigs. This conclusion is in contrast with results from Walk et al. (2012) in broiler chickens and González-Vega et al. (2016) in pigs from 25 to 50 kg. Data from pigs indicated that regardless of dietary concentration of P, reducing dietary Ca to 30% of the requirement reduces gastric pH compared with values for pigs fed diets containing Ca at or above the requirement (González-Vega et al., 2016). However, in the present experiment, Ca reduction was only at 50% of the NRC (2012) requirement, which may be the reason a reduction in gastric pH was not observed.

The lack of differences in fecal score or diarrhea frequency between pigs fed diets with adequate or deficient concentrations of Ca and P reflects the results from stomach pH and further indicates that a reduction in dietary Ca and P failed to change gastric and intestinal conditions. It is possible that this observation is a result of the fact that phase 1 diets used in this experiment contained 15% lactose, which may have resulted in production of lactic acid from microbial fermentation (Suiryanrayna and Ramana, 2015), and therefore, reduced stomach pH, as has been recently reported (Zhao et al., 2021). If that was the case, lactic acid production could have reduced the possibility for a further reduction in gastric pH by reducing Ca and P in the diet. However, it is also possible that the low buffering capacity of ingredients used in this experiment such as corn, soy proteins, and lactose (between 9 and 50 times lower than limestone and MCP; Lawlor et al., 2005), contributed to the lack of differences in gastric pH between pigs fed diets with adequate or deficient concentrations of Ca and P. The buffering capacity of a complete diet is calculated from the buffering capacity of each ingredient (Jasaitis et al., 1987; Lawlor et al., 2005), and as a consequence, the ingredient composition of the diet influences the ability of dietary Ca and P to reduce gastric pH.

Addition of microbial phytase to diets results in reduced need for calcium carbonate and MCP because phytase releases P and Ca bound to phytate in plant feed ingredients (Selle et al., 2009). In the diet containing phytase and deficient concentrations of Ca and P, no MCP was included and the concentration of calcium carbonate was the least among diets. However, due to the lack of differences in stomach pH between pigs fed diets with adequate or deficient Ca and P, it is difficult to conclude that the reason phytase tended to reduce stomach pH is the additional reduction of calcium carbonate and MCP in diets. Nevertheless, the tendency for reduced gastric pH in pigs fed diets with 2,000 FTU of phytase concurs with data from Lee et al. (2018) indicating that inclusion of 2,500 FTU of phytase in diets for weanling pigs resulted in reduced stomach pH (1.81 vs. 2.81) compared with pigs fed a non-phytase diet.

The tendency for a decrease in fecal score and diarrhea frequency in pigs fed diets containing phytase concurs with data from weanling pigs in commercial conditions that had a tendency for increased stool firmness if 2,600 FTU of phytase was used compared with pigs fed diets with 600 FTU (Moran et al., 2017). Therefore, the beneficial effect of phytase on diarrhea occurrence is likely due to its role in reducing the anti-nutritional effects of phytate with a subsequent increase in inositol production (Moran et al., 2019). Gastric pH may increase upon phytase supplementation because phytate has an acidogenic effect in diets due to its ability to bind positively charged amino acids in pepsinogen, which reduces pepsin activity and results in compensatory secretions of HCl in the stomach (Woyengo, 2010). However, data from the current experiment do not support this hypothesis, and more research is needed to determine the influence of phytase and phytate on gastric pH of pigs.

The lack of differences among treatments in serum BUN indicates that protein utilization by weanling pigs is not influenced by dietary Ca and P or phytase. The observed decrease in the concentration of albumin along with the tendency for a reduced concentration of total protein in serum of pigs fed diets with deficient concentrations of Ca and P compared with pigs fed diets with adequate Ca and P is likely associated with the way Ca is distributed in the bloodstream. After absorption, Ca circulates in the extracellular fluid in three forms: free, protein-bound, and complexed. The free ionized form represents 50% of the total Ca, whereas the protein-bound Ca is around 40% of which 80% is Ca bound to albumin and 20% to globulins; and the remaining 10% is Ca complexed with small anions (Taylor and Bushinsky, 2009). Calcium concentration is maintained constant in the blood via hormonal regulation of Ca homeostasis. The kidney is the main regulatory organ, which filters free and complexed Ca, but does not filter protein-bound Ca (Taylor and Bushinsky, 2009). Therefore, even though the level of dietary Ca has limited influence on the concentration of Ca in plasma of pigs (González-Vega et al., 2016), it appears that the levels of Ca and P in diets have an impact on the concentration of albumin in blood. However, more research is needed to confirm this hypothesis.

Results from this experiment provide information about formulation of weanling pig diets based on corn and soy proteins. However, because of interactions among dietary Ca, dietary phytate, and phytase inclusion level (Selle et al., 2009), the outcomes may be different if diets with greater Ca concentration, with ingredients different from corn and soy protein, or with different inclusion levels of phytase are used.

## CONCLUSIONS

Reducing calcium carbonate and MCP in phase 1 diets that contain lactose does not reduce stomach pH or fecal score and has limited impact on growth performance of weanling pigs, but bone development is compromised by Ca and P deficiencies. Lowering Ca and P also results in reduced concentration of serum albumin. However, inclusion of 2,000 FTU of phytase tended to decrease stomach pH and the incidence of diarrhea. Phytase also had beneficial effects on growth performance of pigs fed Ca- and P-deficient diets, which may be a consequence of the destruction of phytate and elimination of its anti-nutritional effects in diets for young pigs.
